# Is adjuvant immunotherapy a new standard for non‐pathological complete response patients with resectable esophageal squamous cell carcinoma?

**DOI:** 10.1111/1759-7714.14641

**Published:** 2022-10-10

**Authors:** Jun Wang, Zhouguang Hui, Qingsong Pang

**Affiliations:** ^1^ Department of Radiation Oncology the Fourth Hospital of Hebei Medical University, Hebei Clinical Research Center for Radiation Oncology Shijiazhuang China; ^2^ Department of VIP Medical Services and Radiation Oncology National Cancer Center/National Clinical Research Center for Cancer/Cancer Hospital, Chinese Academy of Medical Sciences and Peking Union Medical College Beijing China; ^3^ Department of Radiation Oncology Tianjin Medical University Cancer Institute and Hospital, National Clinical Research Center for Cancer, Key Laboratory of Cancer Prevention and Therapy, Tianjin's Clinical Research Center for Cancer Tianjin China

**Keywords:** adjuvant therapy, concurrent chemoradiotherapy, esophageal squamous cell carcinoma, immunotherapy

To the Editor,

Neoadjuvant chemoradiotherapy (nCRT) followed by surgery is a standard of care for patients with resectable locally advanced esophageal or gastroesophageal junction cancer,^1^, ^2^, ^3^ However, 56% of patients still had disease progression or died within 5 years.^1^ Checkmate 577^4^ is a global trial involving patients with esophageal adenocarcinoma (EAC) (71%) or squamous‐cell carcinoma (ESCC) (29%) who did not achieve a pathological complete response (pCR) after surgery following nCRT. Compared with placebo group, adjuvant nivolumab significantly improved disease‐free survival (DFS) (median DFS, 22.4 vs. 11.0 months; hazard ratio [HR], 0.69; 95% confidence interval [CI], 0.56–0.86), which was more obvious in ESCC patients (median DFS, 29.7 vs. 11.0 months; HR, 0.61) than EAC patients (median DFS, 19.4 vs. 11.1 months; HR, 0.75). The results of Checkmate 577 support the recommendations of adjuvant nivolumab for non‐pCR patients after surgery following nCRT, which has been updated in some national guidelines.

In addition to Checkmate 577, two recent studies about adjuvant durvalumab have been published. Mamdani et al.^5^ reported that for patients with locally advanced esophageal and GEJ adenocarcinoma with pathologically residual disease after R0 resection following nCRT, there was a clinically meaningful improvement in the 1‐year recurrence‐free survival rate with adjuvant durvalumab when compared with historical data. However, the result of another study reported by Park et al.^6^ is different from the Checkmate 577 and Mamdani's. This single‐center prospective randomized double‐blind phase II study enrolled the patients with T3‐4N0M0 or T1‐4N1‐3M0 esophageal squamous cell carcinoma (ESCC). After R0 resection after neoadjuvant CRT, 86 patients were randomized 1:1 to durvalumab group and placebo group. The primary and secondary endpoints were DFS and overall survival (OS), respectively. The results showed that there was no significant difference in DFS (HR, 1.18; 95% CI, 0.62–2.27; *p* = 0.61) or OS (HR, 1.08; 95% CI, 0.52–2.24; *p* = 0.85) between the two groups.

Some reasons may explain the different results of aforementioned studies. First, the Checkmate 577 trial was a global phase III study with a higher level of evidence (*n* = 794; ESCC, 21%), whereas Park et al.'s^6^ study was a Korean single‐center phase II study with only 86 patients enrolled. Second, most of the neoadjuvant chemotherapy regimens in Checkmate577 were paclitaxel combined with carboplatin (PC), whereas the regimen of Park et al.'s^6^ study was 5‐FU combined with cisplatin (CF). The PC regiment combined with irradiation might lead to higher pCR rate for ESCC patients (CROSS trial, 49% vs. Park's trial, 31% vs. JCOG1109, 36.7%).^7^ In addition, Park et al.'s^6^ study reported the adequate median number of lymph nodes dissected in the durvalumab group and placebo group (37 vs. 38, respectively), whereas the number was not reported in Checkmate 577. It is seemed that different histological types, neoadjuvant chemotherapy regimens, quality control of surgery, and the difference between programmed cell death protein 1 and programmed death‐ligand 1 (PD‐L1) inhibitors might affect the result of adjuvant immunotherapy.

It is also important to note that Checkmate577 excluded patients who achieved pCR after surgery, whereas Park et al.'s^6^ trial enrolled both pCR and non‐pCR patients, which showed no significant survival benefit of adjuvant durvalumab compared with placebo. Owing to the fact that patients who achieve pCR after nCRT are less likely to develop local regional relapse or distant metastasis, the patients may have less chance of benefiting from adjuvant durvalumab therapy. Moreover, intervention of immune checkpoint inhibitors might increase the risk of immune‐related adverse events. This leads us to think that adjuvant immunotherapy may not be necessary for pCR patients, especially for ESCC patients.

In Park et al.'s study,^6^ the PD‐L1 tumor cell proportion score (TPS) expression after nCRT seems to be correlated with OS and DFS. For patients with positive PD‐L1 expression, durvalumab showed relatively better DFS and OS compared with placebo, although no statistical difference was observed. For patients with negative PD‐L1 expression, the placebo group trend to have better survival. However, in Checkmate 577, consistent trends in the benefit of DFS from nivolumab versus placebo were observed in both subgroups with positive and negative PD‐L1 TPS expression, and in the subgroups with PD‐L1 combined positive score (CPS) expression ≥5 and CPS <5 in post hoc analysis.

Given the positive results of adjuvant nivolumab after surgery following chemoradiotherapy for patients with esophageal cancer, and the negative results of adjuvant durvalumab for ESCC patients, whether the adjuvant therapy of checkpoint inhibitor should be used or not needs to be investigated further. In addition, the current neoadjuvant immunotherapy combination strategy for resectable esophageal cancer is emerging, such as immunotherapy combined with chemotherapy, combined with CRT, and combined with perioperative treatment. However, most of the studies are in phase I to II with small samples.

In conclusion, both Checkmate 577 and Park et al.'s^6^ study have opened up the epoch‐making significance of adjuvant immunotherapy for esophageal cancer patients after neoadjuvant CRT. The neoadjuvant and perioperative treatment for resectable ESCC in the era of immunotherapy still need to be explored further.

## DISCLOSURE

The authors have declared no competing interests.
